# Interrogating green social prescribing in South Wales; A multi-stakeholder qualitative exploration

**DOI:** 10.1371/journal.pone.0314107

**Published:** 2025-01-09

**Authors:** Menna Brown, Katharine Sarah Aylett

**Affiliations:** 1 Faculty of Medicine Health and Life Sciences, Swansea University, Swansea, Wales, United Kingdom; 2 Swansea Community Farm, Swansea, Wales, United Kingdom; Birjand University of Medical Sciences, ISLAMIC REPUBLIC OF IRAN

## Abstract

**Background:**

As an umbrella term, social prescribing offers varied routes into society which promise to support, enhance, and empower individual citizens to take control of their own health and wellbeing. Globally healthcare systems are struggling to cope with the increasing demands of an ageing population and the NHS (UK) is no exception. Social prescribing is heralded as a means to relieve the burden on primary care and provide support for the 20% of patients whose needs are non-medical. As such an increasing array of schemes are available, spanning five sub-sets: creative or nature-based referrals, welfare services, exercise referrals, education programmes or befriending support. Green social prescription offers significant potential to promote wellbeing and improve health outcomes. However limited research has explored this emergent sub-set.

**Aim:**

Explore and interrogate the concept of social prescribing to understand how it is conceptualised, perceived, and experienced by different stakeholders involved in its coordination, delivery, and provision; At a time when it is being formalised in Wales, UK.

**Methods:**

Using qualitative enquiry, from a social constructivist paradigm, stakeholder perspectives pertaining to current social prescribing models, pathways and actions in Wales were explored. Three multi-discipline research workshops and ten semi-structured, one-to-one interviews were conducted either in person or via zoom. Qualitative data were analysed thematically.

**Results:**

39 different stakeholders contributed. These included social prescribers, community connectors, service coordinators, third sector and voluntary organisation representatives, a general practitioner, occupational therapist, social enterprisers, academics and local area coordinators. Five themes were identified which revolved around stakeholders discussions of critical challenges pertaining to the delivery, provision, and evaluation of green social prescribing schemes in south Wales, UK. Tension between varying stakeholders was also evident, often preceded, or complicated by funding discrepancies, competition, and uncertainty. Stakeholders demanded clarity regarding evaluation outcomes and benchmarking across the sector.

**Conclusions:**

To ensure the continued provision of social prescribing schemes which are highly valued by service users, voluntary and third sector organisations require funding security and stability. The delivery of green, nature-based, schemes require maintenance of trusting, long-term relationships with local service co-ordinators and referrers, secure equitable funding models and agreement over conceptual basis of social prescribing itself, particularly in relation to ‘where’ social prescribing is located within health and social care models. Without resolution and positive progress across these areas the continuation of local green schemes within local communities, which build resilience and support positive change for service users’ health and wellbeing, is questionable.

## Introduction

Social prescribing (SP) is a new and rapidly emerging field which holds great promise, to relieve the burden on primary care practitioners and offer structured care pathways which address the social determinants of health i.e., the non-medical factors which shape our lives. Except it’s not new: it’s an innovative rebranding of existing services and informal support systems which have been provided by the voluntary or third sector for a long time (e.g., Bromley by Bow [1]).

### Social prescribing

SP as an approach aims to reduce the burden on primary care in the UK NHS and healthcare systems globally. It is commonly reported that “Around 20% of patients consult their GP for primarily social issues” [2, p6]. As such patients with non-medical needs (i.e. social needs) are referred out of the ‘medical model’ of healthcare, and into existing social prescribing schemes, either directly or via social prescribing link workers, and public sector referral [3, 4]. This approach recognises that health is determined by a range of social, economic and environmental factors, commonly known as the social determinants of health, such as poverty and social isolation and aims to reduce social inequalities and promote health equity [5, 6].

As an umbrella term, SP offers varied routes into society which promise to support, enhance, and empower individual citizens to take control of their own health and wellbeing. Under the umbrella several subsets have emerged: creative or nature-based referrals, welfare services, exercise referrals, education programmes or befriending support [7]. Referrers can link patients to activities across these sub-sets, such as gardening, arts and crafts, or group exercise classes, which provide opportunities for social interaction and engagement [8]. Having stemmed from ‘grass roots’ initiatives significant heterogeneity is evident in the sector, as local communities and organisations develop services in response to local population needs [2].

While there is no current agreed definition, there is general agreement that SP is a systematic mechanism for linking people with existing services and sources of support within the community to help improve their health and wellbeing [9]. Equally social prescribing is understood to be a complex intervention, based on several key principles, including person-centred care. Working in partnership with patients [10], social prescribing link workers (SPLW), identify patients’ goals, aspirations and unique circumstances and develop personalised care plans that reflect individual needs and preferences [8]. This model also draws on a community and asset-based approach to health promotion [11]. SPLWs connect patients with local community services. Finally social prescribing adopts a collaborative approach; it is viewed as a partnership with and between stakeholders, including patients, healthcare professionals, community organisations, and local authorities [12]. It is important to note that social prescribing is not considered an emergency or acute service [13].

Globally 17 countries have a recognised SP agenda [13, 14] including England (UK) [15]. In Wales (UK) a national framework was announced in December 2023 that builds on multiple Welsh government policies which have consistently focused on supporting citizen health and wellbeing and articulated a solid commitment to a preventative approach to health. For example, the goal of the Future Generations Act (Wales) 2015 [16] “*is a society in which people’s physical and mental well-being is maximised and in which choices and behaviours that benefit future health are understood*”. ‘A Healthier Wales’ [17] voices this commitment.

### Evidence

While political agendas have embedded SP into healthcare services, the evidence base remains mixed. For example, systematic reviews have widely reported positive effects of participation, including the establishment of new friendships and social links, a sense of belonging, reduced social isolation, increases in service users’ ability to take action to improve their own health and wellbeing, improved sense of social connectedness, reduced healthcare utilization, and improvements in meaningful employment and return to work rates [18–24]. Many of which have also reported that community-based schemes are well-received by patients and healthcare providers alike [21]. However systematic reviews have also consistently reported key limitations, such as small-scale studies, high risk of bias, lack of comparison, short follow-up periods [22–24] and no benefit for GP workload [25] despite noting many positive outcomes including improvements in service users’ self-esteem, self-value, and hope.

Looking at nature-based social prescribing (NBSP) specifically several studies have reported positive outcomes for service users. One green SP programme provided by Gloucestershire Wildlife Trust [26] reported significant levels of increased wellbeing for patients recovering from cardiovascular health conditions. Two green SP projects reported positive value, post pandemic [27, 28]. And a 12-week outdoor walking and climbing scheme in North Wales showed a positive financial return on investment; for every £1 invested, up to £5.36 was generated [29]. Thus, effectively contributing to national policy goals and improved health and wellbeing outcomes at individual level. Several recent reviews have also added support. For example, one American review [30] concluded that NBSP in urban settings improved social connectedness. However, authors noted additional research was required to understand the impact for at-risk populations. While a review of 38 studies [31] reported that group based, NBSP cultivated social connectedness with greater evidence for longer term interventions. Furthermore, benefits of NBSP are reported for mental health outcomes when combined with traditional therapeutic approaches [32]. While a large three-year UK evaluation study concluded there was clear evidence that NBSP leads to positive impact on people’s mental health and wellbeing.

However, as NBSP emerges and expands it is imperative to understand not just the service user perspective but also the service coordinator, referrer and service providers experience and perspective i.e., a multi-stakeholder perspective is critical. Without referrals into NBSP schemes and without provision of NBSP schemes, the approach will fail. As such the current study aimed to interrogate and explore the concept of social prescribing from a multi-stakeholder perspective, to develop understanding of the systems and services in place at a local level and the views and experiences of those currently involved. Qualitative enquiry enables in-depth examination and exploration [33].

## Materials and method

### Ethics

Ethical approval was obtained from Swansea University Medical School Research ethics committee (ref# 2022 SUMS RESC 2022-0016A).

### Research objective

The study set out to answer the following question ‘how do different stakeholders conceptualise and perceive social prescribing, in South Wales (UK) with a focus on NBSP?’

### Approach

A qualitative approach was used. A series of sequential group-based research workshops were facilitated to enable discussion and interrogation of the concept of SP and the local mechanisms in place to co-ordinate and deliver SP, from a variety of viewpoints; supplemented with one-to-one interviews to explore group identified issues in further depth and detail [33].

### Recruitment

Local stakeholders currently involved in the co-ordination, referral and or delivery of NBSP initiatives in South Wales (UK) were identified through document review, review of local council internet pages, existing network contacts and through discussions with staff at one local NBSP scheme (Swansea Community Farm; who offer a structured programme of outdoor volunteering targeted at adults with mental health issues, long term unemployment and other disadvantages). Identified stakeholders were invited via email to take part in a series of research workshops. Those emailed were encouraged to share the invitation with relevant contacts, colleagues, and networks, to ensure as wide a reach as possible.

This purposeful, convenience sampling approach identified a range of stakeholders; including those who coordinated SP services in the local area, acted as link workers, provided SP schemes, and several social enterprisers involved in the creation of community schemes and academics involved in local SP projects. It was anticipated that each workshop would include 15–20 stakeholders of which, a range of stakeholder roles would be included, to ensure discussion from different perspectives and to enable networking and information sharing.

### Participants

Participants were NBSP stakeholders in south Wales (UK). Inclusion criteria were

1. Actively involved in the co-ordination*, referral** or delivery*** of NBSP services in south Wales* and wider stakeholders****
2. Aged 18 and above.
3. Ability to provide informed consent.

*Service co-ordinators, defined here as those coordinating the delivery and funding of SP schemes in the local area.

**Service referrer, defined here as individuals working in a link worker role who had responsibility for patient referrals into a NBSP service/scheme.

***Service providers, defined here as those who are funded to facilitate and run the actual NBSP schemes that patients are referred into (form any source including self-referrals)

****other interested parties for example, academics and researchers, social enterprisers.

### Data collection

Data were collected via three research workshops, and ten one-to-one interviews held between February and July 2023.

The first and third research workshops were held online using zoom technology and these were audio and video recorded and professionally transcribed. The second was held in-person at a local NBSP venue, Swansea Community Farm, a small city farm. The workshops produced group notes, recorded on flip charts and researcher field notes and observations.

All workshops included an initial welcome and project overview. However, each workshop was designed as a standalone event, to ensure a variety of stakeholders could attend. The workshops focused on exploring stakeholders’ understanding of SP, current models and practices in place locally, and the role of NBSP schemes in Wales in relation to current Welsh public policy. At each workshop two different stakeholders were invited to present and discuss their NBSP schemes which was followed by a question-and-answer (Q&A) section. This also provided an opportunity for knowledge transferral. After the presentations and Q&A the researchers introduced a specific topic for discussion as a stimulant for the remainder of the session. Workshop topics were: ‘what does SP mean to you?’ ‘Metrics, measurement and impact’, and ‘organisational needs’.

At the end of each workshop a summary and thank you speech was included. At which point all participants were invited to take part in a follow-up one-to-one interview, the purpose of which was to explore topics covered in the workshop in further detail and depth; to seek clarity on topics raised and find out more about individual stakeholder roles. The subsequent interviews were facilitated [MB] via zoom or in-person, and these were arranged via email and scheduled for a mutually convenient time and date. Each was audio or audio, and video recorded, and professionally transcribed for analysis.

### Data analysis

Data were analysed using reflective thematic analysis informed by the work of Braun and Clarke [34]. This involved following a staged process; stage one involved re-familiarisation with the data collected via rereading of the transcripts and revising the flip charts, and researcher field notes. Stage two involved line by line coding of transcripts [MB] using inductive coding which informed the creation of a thematic map. This was then used repeatedly by the researchers to aid discussion of the data. For example, the thematic map helped the researchers to organise the data, to identify patterns in the data; this included noting reoccurring topics, issues and points of interest raised by the stakeholders and identification of areas of agreement or disagreement. The codes were mapped and grouped to identify themes. Themes were agreed and labelled and associated codes and raw data were re-examined for validity. Themes were then shared with stakeholders for commentary via email before being finalised.

## Results

### Participants

In total 39 different stakeholders took part. A total of 55 invitation emails were sent ahead of workshop one, 12 of which generated interest, but recipients indicated that the date was unsuitable and 21 agreed to attend. A further 24 contacts were identified ahead of workshop 2 of which 15 responded to say they were interested but the date didn’t suit and 16 agreed to attend. However, one did not attend on the day. The final workshop had the least attendees, only 7 stakeholders were present, four of whom had attended a previous workshop. No additional contacts were identified and invited ahead of this workshop. Table 1 reports the breakdown of participants per data collection activity and reports the date, delivery format and type of stakeholder included. The variety of stakeholders included in each workshop meant that a range of different NBSP schemes were included for discussion across each workshop as planned.

**Table 1:** Workshop and stakeholder descriptions.

Data collection	Date	Format	Total number of participants	Stakeholder roles
**Workshops**				
Workshop 1	7.2.23	Online via zoom	21	Social prescribers
				Third sector organisation representatives
				Early career researchers
				Academics
				Social enterpriser
				*WSSPR*
Workshop 2	17.3.23	In person	15	Social prescribers
				Third sector organisation representatives Local area coordinators (LAC)
				Service coordinator
				Community connector
				Early career researchers
				GP
				Occupational therapist
				Health economist
				Social enterpriser
Workshop 3	19.4.23	Online via zoom	7	Social prescribers
				Early career researchers
				Academics
				Health economist
				Social enterpriser
**Interviews**				
1	10.2.23	Online	1	Third sector organisation representative
2	26.4.23	Online	1	Community connector
3	5.6.23	Online	1	Social prescriber
4	9.6.23	In person	1	Third sector organisation representative
5	9.6.23	In person	1	Third sector organisation representative
6	9.6.23	In person	1	Third sector organisation representative
7	22.6.23	In person	1	Service coordinator
8	22.6.23	In person	1	Social prescriber
9	18.7.23	On-line	1	Local area coordinator (LAC)
10	26.6.23	On-line	1	Local area coordinator (LAC)

### Social prescribing models

**Fig 1:**
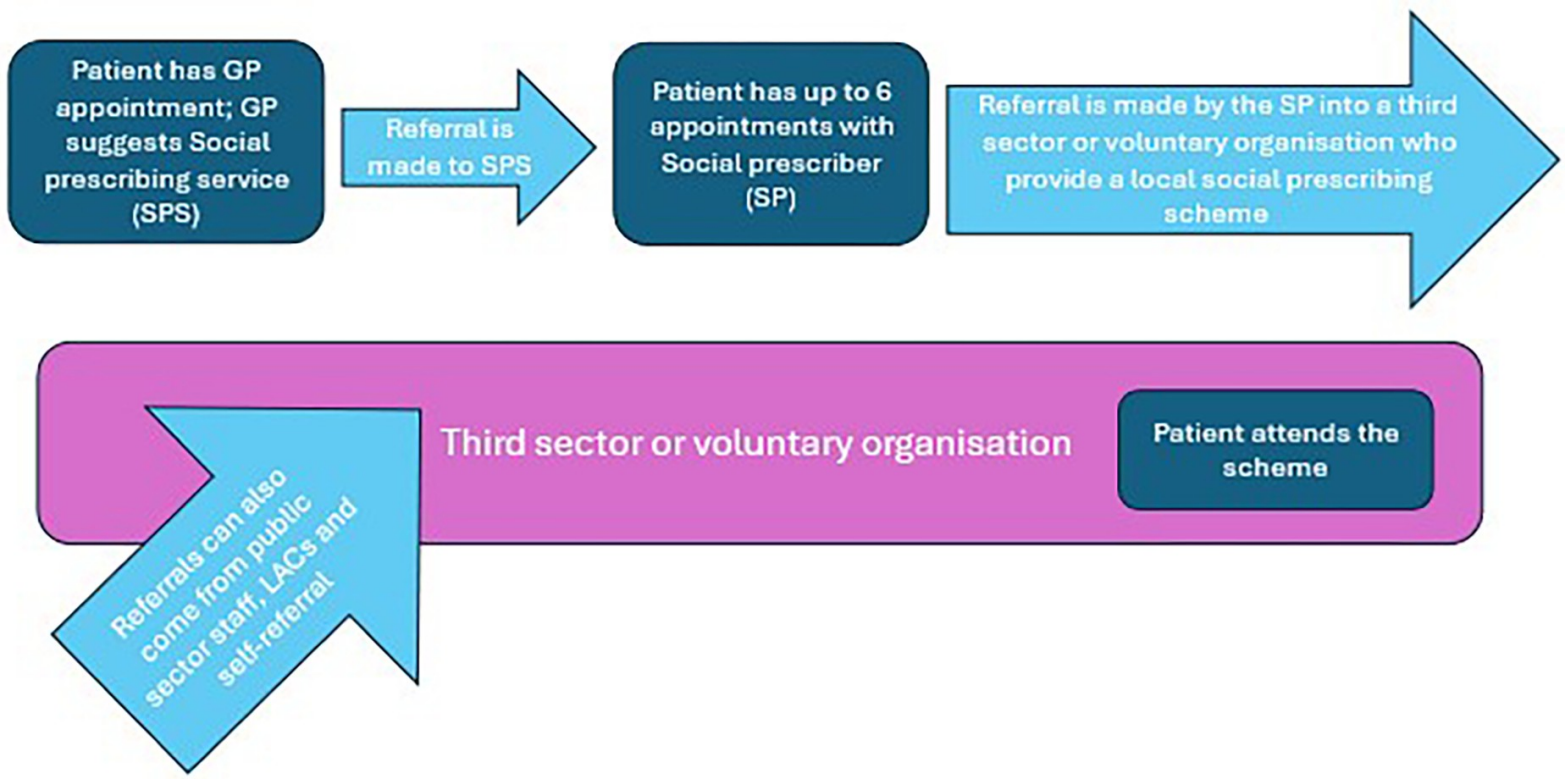
Flow chart depicting the flow of patients into SP schemes in the local area.

The workshops identified several different approaches to SP and these align with SP models described in the NFSP [7]. For example, the ‘social prescribers’ and ‘community connectors’ role align with the ‘SP link worker model’ whereby GPs and other primary care professionals refer patients into a coordinated social prescribing service, (see Fig 1) often based in GP clusters. For example, in the local health board area five ‘social prescribers’ offered services for a duration of 17.5 hours per week each across five GP clusters which covered the geographic range of the local health board, which until March 2023 was a virtual only service (due to COVID-19 pandemic) and which was slowly returning to an in-person service. Taking a person-centred approach these ‘social prescribers’ described their role as involving the identification of individual needs and the co-production of a personalised social care plan. They provided individual sessions which lasted between 30–60 minutes. Depending on geographical area, the number of sessions offered varied, in the local health board area it was up to six with an additional six available where need arose. In a different health board, only three sessions were available. These social prescribers offered supportive conversations, life coaching (however this has recently been removed from the role), signposting to ‘basic needs’ support services (which included, housing, employment, and financial support) and or transferral into therapeutic mental health services (at which point the patient exited the SP service). During the sessions, onward referral into third sector organisations was often made, based on individual interest, capacity and availability.

Other link worker roles identified were called community connectors. These individuals undertook a similar role, they supported individuals and made onward referrals, but their service was accessible independently of primary care.

Across Wales, the terminology used for social prescribers varied considerably. Table 2 shows a list of terms identified during the data collection phase. The Wales School for Social Prescribing Research (WSSPR) have published a glossary of terms to provide clarity and to standardise the language used within the field [35]. This may help address the confusion apparent in the field.

**Table 2:** Social prescribing link worker titles and areas identified in use.

Name used	Area	Funding streams	Clusters where link workers were based
Community connectors	Pembrokeshire	LEADER, Pembrokeshire County Council, Hywel Dda Health Board, Welsh Government (ICF funding) and Volunteering Matters	4 GP practices
	Hywel Dda University Health Board, Llanelli	LEADER, Pembrokeshire County Council, Hywel Dda Health Board, Welsh Government (ICF funding) and Volunteering Matters	4 connectors
	Powys Teaching Health Board	*	10 Localities
	Cardiff and vale health board	MIND, NHS,	3 GP cluster locations
Health and wellbeing co-ordinators	Powys Teaching Health Board	*	*
Social Prescriber / Resilience Worker	Hywel Dda University Health Board, Llanelli	*	5 GP practices
Community Coordinators	Cwm Taf health board	Integrated Care Fund and are hosted by the County Voluntary Councils, Interlink & VAMT.	5 Coordinators
General Practice Support Officers (GPSO)		*	*
Practice based Lifestyle Champions		*	*
GP wellbeing coordinator	Rhondda Valleys	*	12 GP practices
Social Prescribing Co-ordinators	Neath Port Talbot	Mental Health Service Improvement Fund	8 clusters
Social Prescriber	Swansea Bay University Health board	SCVS	5 clusters
Social Prescriber	Aneurin Bevan University Health Board	County Borough Council	2 clusters
		Intermediate Care Fund	
Community Wellbeing Coaches	Cardiff and Vale (Central Vale)	Communities First	*
ACE prescription			*
Wellbeing 4 U Coordinators		*	2 GP hubs
Individual SP projects		*	Variety
Community link officer	Betsi Cadwaladr University Health Board	Fixed term Cluster funding	2 GP Practices
Community Facilitator		Intermediate Care Fund (ICF) committed until 2017	*
Community Navigator			*

*This information was not identified in the research workshops, reported online or was not publicly available

The second model identified was the local area coordinator (LAC) model; funded by the local council and supported by additional grant funding this takes a long-term approach to supporting people within the community and is not time bound. For example, clients (not patients) are supported for as long as there is a need. In one example provided covered a three-year period. In the local health board area 23 LACs are embedded in the local community. LAC stakeholders described their role as ‘*walking alongside clients’* (interview 9, LAC) taking a strengths-based approach. They initiated a connection directly with clients within their community or via informal and formal connections [36, 37]. LACs also made onward referrals into third and voluntary sector SP schemes including NBSP schemes and had a dual role to support the development of community schemes and facilitate social connections and the development of social capital.

In addition to these two models, citizens can self-refer directly into third sector or voluntary SP schemes, and they can also request to see a SP via a GP receptionist (if the surgery offer SP) or they could be referred by public servants e.g., (social workers, Occupational therapist, librarians, allied healthcare professionals etc.) who have a civic duty to upload the values of the future generations Act [14]. Both these referral routes are recognised in the newly announced national framework for SP [7].

### Thematic findings

Five themes were identified from across the research workshops and interviews, three focused on current barriers and challenges faced by stakeholders and two focused on uncertainty regarding evaluation and monitoring efforts to capture effectiveness and value of schemes for service users. Service coordinators and referrers did not comment on these latter two themes. The themes were *‘Lack of consensus*’, ‘*Tension’*, *‘Funding’*, *‘Evaluation’* and *‘Uncertainty’*.

#### Theme 1; Lack of consensus

A general lack of consensus or disparity in views regarding social prescribing was observed during the workshops and interviews. Stakeholders disagreed about what ‘counted’ as SP. For example, whether self-referral or referral from outside of primary care was considered SP. In contrast others considered ‘all’ opportunities where people entered SP schemes as ‘counting’.

*‘Is it social prescribing if the referral isn’t from a health prof? Surely that’s what social prescribing is all about? …if they are being referred by other agencies then that’s great but surely that’s not SP or if it is SP is everything. It just seems a bit strange, the word prescribing links it to the health service (workshop 1, third sector representative).*

‘If you recognise that your health can be improved by taking part in a particular activity, and you make this happen without a social prescriber, i would say it’s still social prescribing’ (workshop 1, third sector representative).

*“It’s an umbrella term…there seems to be confusion around referral and self-referral…the national framework mentions a model that defines self-referral and we’ve produced a flow chart that shows the pathways as well” (workshop 1*, *WSSPR)*.

This lack of consensus extended to the location of SP, should it belong in the healthcare system, the third sector, or local authority? Currently it exists in all three locations and debate included disagreement about the medicalisation of SP and resultant implications for funding (see Theme 3).

*‘Should Social prescribers be part of the NHS, Local Authority or Third Sector–where do they belong?’ (workshop 1, third sector representative)*

‘we see it as finding ways to support people without it being a formal setting’ (workshop 1, third sector representative)

*‘Social prescribing can be a model for inclusivity*, *including rural and BAME communities*, *it should be an integrated service*, *not based in health’ (workshop 1*, *Social enterpriser)*

In line with this disparity, the LACs did not consider themselves to sit within the SP framework.

*‘it’s very different from lots of other statutory organisations or third sector, in the way that we’re based on some values and principles that are very much about doing with people rather than doing to…we don’t call them referrals we call them introductions, because language is important’ (interview, LAC 2)*

*‘I think we’re very different from social prescribing’ (interview*, *LAC 2)*

In addition, stakeholders queried what SP consisted of, conceptually; is it a person, a place or an activity?

*‘Does SP have to be an activity–can it be a place? (workshop 1, third sector representative).*

*‘I feel it spans the bio-psychosocial model not one that starts in health and is sent out…*.*instead of a medical therapeutic prescription it might be Mrs jones needs to get involved with this but to do that she might need childcare and she’s a wheelchair user so she needs support for two hours to enable her to attend a support session*. *That’s the social prescription’ (workshop 1*, *social enterpriser)*.

Stakeholders raised concern about this apparent disparity and lack of common understanding. *‘I’m worried that people fall through the gaps between services’ (workshop 1*, *OT)*. However, many noted that the incoming national framework might provide clarify going forwards.

It was also apparent that staff within third sector and voluntary organisations often supported patients in the same way as the social prescribers, community connectors and LACs did. Specifically, they supported individuals to access housing, financial and employment services, signposted and made onward referrals themselves alongside providing the SP programme in question. As such some third sector organisations staff viewed themselves as ‘social prescribers’.

*‘We often support our volunteers to access ‘basic need’ services, and we also make onward referrals into other third sector organisations or into local communities schemes via the LACs’ (interview, farm representative).*

However, this additional support was often restricted to larger third sector or voluntary organisations, due to capacity, and skills available. Stakeholders from these organisations noted several limitations of their schemes which restricted their ability to accept some citizens. For example, *‘we have had referrals of people with additional needs and we are not set up for that*, *with volunteers in charge*. *We can provide SP but not for people with complex needs’ (workshop 1*, *third sector representative)*

#### Theme 2: Tension

There was tension between service co-ordinators, service referrers and service providers across several topics: referral into NBSP schemes, overlapping roles, and funding. For example, during the workshops third sector stakeholders questioned how social prescribers identified, selected, and referred patients into NBSP schemes. Noting that they believed social prescribers were often reluctant to refer patients into their individual NBSP initiatives. They compared this to referrals made into other types of SP schemes like arts based-creative schemes or exercise and lifestyle schemes and befriending schemes; the assumption made being that patients were not often sent to NBSP schemes. In some instances, third sector organisations reported they were under subscribed as a result. Equally they wanted clarity of how referrals were decided.

*“Its not working- referrals not getting to the organisations. Coordinated approach is missing” (workshop 1, third sector representative)*

‘There seems to be a collective reluctance to engage with SP someone needs to step in and say let’s make this work…There is something fundamentally wrong with the system.’ (workshop 1, third sector representative)

*“We got funding through the RDP to run a pilot project called ‘wellbeing through woodcraft’*… *It came as a massive surprise to realise that we just couldn’t get people to participate and it looks like a large percentage of that money isn’t going to be spent now…I see it as a great opportunity but there is something missing between that linkage between GPs*, *health professionals and people like myself and (third sector representative*, *name) who deliver these activities*, *I’ve been ready and waiting*, *but something is not working and that’s what needs to be explored now” (workshop 1*, *third sector representative)*.

How would we interact with you [SP] to make referrals happen? (*workshop 1*, *third sector representative)*

“*We want to offer SP opportunities, but it’s not clear how different organisations’ (GP cluster, SPLWs, LACs) link up’ (workshop 1, third sector representative)*

In response social prescribers reported challenges associated with onward referral into NBSP schemes which included, a client base who they described as ‘not ready’ to access outdoor activities; ‘not ready’ to connect with others via group-based community- programmes. Equally they believed that the opportunities provided by NBSP schemes were not always relevant or appropriate for some of the populations that they served. For example, the elderly, and those who are house bound or who had social anxiety. They suggested only 10% of the people they saw in their service were ready for green NBSP opportunities. Finally, clients who do not disclose an interest in nature-based activities were also not offered the opportunity.

“…[we] *talk through a series of things that are happening in their [patient] lives and we carry out research based on what’s happening to them and then it’s really led by the individual and they really make the decisions on how to move forward in their lives* (workshop 1, SP)

“…*current state of NHS mean we are absorbing some of those issues into SP*, *a lot of referrals coming in are awaiting packages of care or input from social services…*. *They aren’t suitable to refer into a forestry project or something like that because what they are dealing with*, *needs to be dealt with first*. *Another issue is because we are coming out of the pandemic*, *there is a lot of anxiety about attending project*, *they are tending to go to regular lower-level things like wellbeing walks held in in the park every week rather than e aging in learning things or something more adventurous or ones that involve a more social aspect*” (workshop 1, SP).

“*Another thing in my cluster is I get a lot of elderly house-bound referrals so again they are not suitable to be referred to outdoor activities*, *equally we have a lot of students who are not registered with GPs*” (workshop 1, SP)

“*Very high anxiety levels…might not have the finance or confidence to access things outside their area*” (workshop 1, SP)

However, they did acknowledge that while they were required to stay abreast of the varied third sector services, it was often difficult to do so, due to the time consuming nature of the task, made more complicated by the lack of an up to date database; notably due to limited staff funding and the impact of funding changes on existing SP schemes i.e. they ran for a specific time period and ended, new ones were funded and older ones vanished. As shown in the following extracts.

*‘Part of my role is to do very comprehensive mapping of what’s available in the community…its not even the cream on the cake it’s a main ingredient the third sector’ (workshop 2, SPLW)*

‘*no one list of third sector organisations to refer to’ (workshop 1*, *third sector representative)*

As such third sector stakeholders called for transparency, standardisation and clarity surrounding the referral processes from referrers. Both discussed these tensions in relation to the demands on their time required to establish, cultivate and develop relationships to support referrals, only to see them repeated due to high staff turnover and limited availability of community schemes.

#### Theme 3: Funding

The tensions articulated between stakeholders were raised at each workshop. The primary feature being the growing reliance on third sector, voluntary organisations to provide services to increasing number of citizens, with increasingly complex needs, without financial government reimbursement. Specifically, third sector organisations noted that they are required to obtain funding from non-governmental awards such as national lottery funding to provide their NBSP services. Many believed they should receive healthcare funding from NHS Wales or from local councils. This tension was made worse by disparity in funding arising from the location of some SP programmes within the Healthcare sector and others outside of it. For example, many arts based, and creative social prescriptions are funded by NHS Wales. Equally some NBSP schemes are funded by service pco-ordinators e.g. MuD (Minds under Development) an outdoor mindfulness and forest bathing scheme offered in the local health board, designed to promote well-being, reduce stress and boost the immune system. The key issue being that those within the healthcare sector received central (government) funding. This meant that the majority of third sector organisations were reliant on charitable donations and competitive often short-term grant funding. The implications being threefold; valuable, effective and well managed programmes regularly disappear from the SP landscape making it difficult to provide long term structured, standardised services for those in need. Meaning that social prescribers had to proactively and regularly update both their knowledge and databases of local schemes, and finally third sector staff turnover and job security was uncertain.

*‘The voluntary sector is taking on people who should be publicly funded, if it’s replacing medical treatment, it should be public sector funding. Short-term funding means that there is no consistency or security [in the sector]–causes problems with recruitment. The focus on numbers by funders is frustrating–doesn’t recognise that some people with complex needs will need more time/support’ (workshop 1, third sector representative)*

‘the problems we’ve got is a lack of funding (workshop 1, third sector organisation)

‘We are all scrabbling around for funding, the same pots of funding, we are going to be squeezed in the future. So, working together will be critical’ (workshop 2, third sector)

*‘They are giving less and less’ (workshop 2*, *SPLW)*

Despite health professionals, including those in our workshops, recognising the value of NBSP for their patients, there was little experience of direct funding from NHS Wales. One scheme ‘Grow Cardiff’ received direct funding from their local GP cluster but relied on grants for the majority of their income. However, they noted the need to tailor language used for the healthcare context to develop understanding across organisations to achieve this funding in the first place. In the local area GP clusters offered funding via a Local Cluster Collaborative Mental Health Intervention grant scheme, which invited service providers to apply. Participants who had received this funding felt their organisations had benefited, but it did not allow their organisation to move away from reliance on grant funding and so were still trapped in a short-term cycle of service provision. Equally they had not received as many referrals as anticipated from the GP cluster they were aligned with. Although they did not comment on whether funding was reliant on numbers of patients referred and or attending.

#### Theme 4: Evaluation

A common thread of discussion across the second and third workshops was the importance and role of evaluation to demonstrate effectiveness to secure onward funding. Third sector organisations considered the tools they used, the ability of service users to engage with the tools selected, and the sheer variety of tools available which limited meaningful comparisons of outcomes across the sector. Stakeholders expressed interest in identifying a gold standard tool. Ability to evaluate and show value for money also fed into stakeholders’ interest in transparency in referral routes. Standardised outcomes would enable meaningful comparison across local schemes and enable transparent fundings decisions based on empirical data.

*‘People are looking for benchmarks which we can meet–the 3rd Sector wants to provide the standard expected by the health board, GPs, etc, but we don’t know what that is’ (interview, third sector representative)*

‘we need accurate monitoring to prove value of service to GPs etc’ (workshop 2, third sector representative)

*‘I want to know what structures we need to follow…we have a staffing structure*, *a development plan that staff go through*. *For me it’s being aware of what other organisations want’ (workshop 2 third sector)*

#### Theme 5: Uncertainty

A final thread in the discussions centred around stakeholders’ uncertainty. The national consultation [3] was completed three months prior to the project being undertaken, some stakeholders had been involved and others had not. Discussions focused on the emerging formality of SP nationally and a sense of anticipation was observed as to how this would shape and change provisions in place. There was uncertainly in regard to how roles may change, how ways of working might need to be updated to standardise practices nationally and the need for training and resources to implement this at organisational level.

*‘Its [SP] here to stay but we need to be flexible as the model is changing’ (workshop 2, SPLW)*

‘Is SP being formalised in the right way—where should SPs sit? Moving from NHS to LA in Llanelli—‘surely they sit better in health?’ But community connectors sit better in 3rd Sector, because not politicised—politics can get in the way. But then funding is an issue. Who is covering young people? SPs deal with 18+? (workshop 2, third sector representative)

*Is the framework even helpful*? *(workshop 2*, *third sector representative)*

One social prescriber (from a different health board to the local area in question) noted how their role has already been reduced and the pay awarded changed to reflect the removal of professional autonomy to ensure consistency in delivery (Interview 1).

Finally, of note, all stakeholders involved in the study had positive views of the schemes they co-ordinated, referred into and provided and many offered ideas on how to develop local services to maintain momentum and ensure support provided continued improving and developing for the benefit of service users.

‘*it’s the cavalry [the third sector] not the public sector (workshop 2, SPLW).*

## Discussion

The research workshops, and interviews created a shared space to discuss, explore and interrogate the concept of social prescribing. The objective was to understand, from multiple stakeholder perspectives, how SP was conceptualised and perceived at a time in Wales, UK when a national framework was imminent.

Findings highlighted several important topics which have bearing on how social prescribing is accessed, funded, and ultimately delivered, to its myriad of (often) vulnerable service users. Service users living with disability, poor mental health, and disadvantage and who rely on non-medical services to improve their quality of life. It identified issues of concern for those involved in its coordination and delivery and shed light on the intersection of funding and conceptualisation.

The qualitative analysis undertaken identified five themes: the first focused on conceptualisation of SP and three highlighted issues of importance to stakeholders and showcased the challenges currently being faced by those in the sector. The final theme reflected service providers’ narratives on the uncertainty they faced within the sector and likely was in part related to the current changes that are under way in Wales in relation to SP.

The lack of consensus on how SP was conceptualised by stakeholders was of interest. Different stakeholders held different ideas about what ‘counted’ as SP, based on entry points (referral) to SP programmes. Stakeholders did not agree on how support should be delivered. This varied per SP Model for example, social prescribers saw their role as short-term support which involved helping patients to access ‘basic services’, signposting to statutory services, and onward referral into third sector schemes including NBSP schemes (or other SP sub-sets), and community connectors saw themselves as life coaches who supported people to enter community schemes providing SP services. In contrast LACs saw their role as a long-term, values-based approach, to building community resilience and social connectedness where they also supported people to access SP schemes.

Equally, there was disparity in opinion between stakeholders’ views on where SP should be located. Specifically, whether such services belong within the medical model of healthcare, accessed via GPs and primary care-based health care professionals (who make a referral to a link worker or directly prescribes an SP activity). Or whether it belongs outside of healthcare system entirely. Discrepancy in how SP is conceptualised has implications for how it is operationalised in south Wales and potentially beyond. Where there was agreement was in the purpose and value of SP for those in need of non-medical support, and individual representatives from third sector NBSP providers discussed their collective agreement on what was offered and afforded by such schemes.

Several themes highlighted current challenges faced by stakeholders, for example, it was evident that there was tension between some service co-ordinators (i.e., coordinating bodies some of whom had funding responsibilities), referrers, (i.e. social prescribers (who sat within the co-ordinators organisation, and LACs) and service providers (i.e. third sector voluntary organisations) which stemmed in part from the mismatch of views relating to the conceptualisation of SP, and funding concerns. Specifically, third sector organisations felt that referrers did not know they operated schemes locally, did not make adequate referrals into their schemes and were not supportive of NBSP generally. The funding issue complicated this further as, in some instances, they were in direct competition with each other for financial support. Although in the main third sector organisations were funded via different streams: some services received central government funding (NHS funding or local authority) which afforded longevity and security whilst others were plagued by short-term highly competitive charity sector grant funding which reduced staff capacity to deliver the on the ground services, valued by service users.

The issue of funding was widely discussed by service providers and sometimes acknowledged by referrers, although all stakeholders recognised the impact of the growing reliance on such organisations to provide schemes, to lessen the financial and capacity burden currently being experienced in NHS Wales (and England). It will remain to be seen if the new NFSP will address these concerns. Stakeholders noted uncertainty surrounding metrics required for evaluation. For example, service providers discussed tools they used to evaluate their schemes and how they reported their outcomes which were in turn used to secure onward fundings and demonstrate effectiveness and value for money. Here the tension arose again as some third sector organisations’ representatives expressed the need for transparency in referral decision making. They expressed a need to clarify industry standards, procedures and expectations of organisations to enable meaningful comparison between NBSP programmes which in turn they assumed would be reflected in referral and funding decisions. Their discussion also centred on the need to identify specific tools for evaluation which could be used across schemes to further aid comparison and transparency. However, research has offered recommendations for evaluation [38] which could inform stakeholder decisions. Finally, service providers openly discussed their uncertainty about the future of SP; some referrers also noted how they had already seen their roles, responsibilities and pay impacted by national changes.

All of this uncertainty, tension and worry was set against a backdrop of service user gains [39]. Previous research has demonstrated the value of third sector NBSP provision, specifically opportunities to volunteer outdoors, to society, and to the health services. The Wildlife Trusts’ Social Return on Investment report found a value of £6.88 for every £1 invested for people with poor mental health taking part in a ‘nature on prescription’ programme [40]. Research into programmes run by the Outdoor Partnership found a SROI of between £4.90 and £5.36 for every £1 invested [41] and more recently a social return on investment of £2.42 for every £1 invested was reported for the prevention of mental ill health [39].

### Comparison to literature

We are unaware of similar studies which explored stakeholder views of SP which revealed or discuss a lack of consensus on how SP is conceptualisation. However, we are aware of the confusion in the sector resulting from the myriad of terms, roles and descriptions used to explain and describe those involved in its coordination and delivery. The SPLOSSARY [35] and NFSP [39] will be of value in addressing these differences.

When considering the themes identified in the current study, many are in line with recent literature. For example, Bertotti et al. [42] reported a realistic evaluation of a green SP intervention to address poor physical health. Authors identified that while social prescribing had benefits for individuals’ communities and society, key issues for service providers remained unaddressed. Those issues resonate with the findings of the current study. Namely the critical need for long-term, sustainable financial support for third sector voluntary organisations who provide the NBSP activities which GPs, SPLWs and LACs (and others) refer into. Secondly, they discussed the need for ‘buy in’ from primary care refers such as GPs to ensure visibility and increase service accessibility within the medical model. This was also a critical challenge for social prescribers (referrers) and third sector organisations (providers) alike. An issue which took valuable service time and required significant investment in terms of time, relationship building and resources. Of interest the one GP in attendance was aware of SP but did not work directly with the five social prescribers in the health board.

Analysis was also in line with qualitative findings reported by Fixxen & Barrett [27] who outlined critical challenges associated with green social prescribing in the context of the Covid-19 pandemic. For example, their data also indicated that stakeholders had critical concerns relating to longevity of funding, sustainability of schemes and, widespread agreement that social prescribing link workers were hesitant to refer into NBSP interventions due to worries about relevancy, age and accessibility. Equally Sumner, Sitch, & Stonebridge [26] reported similar barriers to engagement which included mobility and fitness concerns and differing priorities. While McHale et al. [43] reported similar stakeholders’ (n = 55) concerns relating to sustainability of funding in Scotland.

Improving population health requires SP to be comprehensive and to address domains beyond the traditional medical model to include health promotion and the social determinants to impact the entire population: it needs to be universal and accessible to everyone. Fragmentation in care means that those less able to manage their own health are more likely to ‘fall through the gaps’, and thus, integrating SP into the healthcare system can improve accessibility and navigation through services systems are integrated, they have an increased ability to adapt in tandem with other services [44].

### Study limitations

While this study explored stakeholder perspectives using qualitative data collection methods the findings reported here should be interpreted in the context in which they were gathered. Equally they are time bound. They represent views of the current system and models in place in Wales, UK and the benefits and challenges highlighted may change over time. As noted earlier, the study was conducted prior to the Welsh government publishing their national framework for SP and thus stakeholder perceptions are likely to change following the formalisation and standardisation of the services in place. As such, future work should continue to explore multiple-stakeholder perspectives to understand and address sector challenges following the implementation of the framework to understand the impact this has had on stakeholders as well as service users, not to mention society. It would be valuable to return to these stakeholders and find out how the national SP framework has impacted their roles and organisations and whether funding challenges for example have been alleviated.

## Conclusion

This study explored via qualitative methods, multiple-stakeholder perspectives pertaining to social prescribing in South Wales, UK with a focus on nature-based social prescribing. It reports several challenges facing the sector which need to be addressed to ensure effectiveness and sustainability of SP services.

The third sector plays a critical role in contributing to a prosperous Wales and meeting the seven wellbeing goals outlined in the future generations act (2015) Wales [16]. However, voluntary and third sector organisations reported significant concerns regarding funding, tension and uncertainty which impacted short-term and long-term delivery of green social prescribing schemes of value to service users. This issue must be addressed at a national level if reliance and development of SP is to be realised, as increasing reliance on providers outside of the medical model of care are demanded; if the aim to relieve the burden on primary care is to be realised. The findings have implications for service provision, relationship and capacity building at local and national level which limits service provision; services valued by those who access them.

Equally attention needs to be paid to supporting and standardising evaluation of nature-based social prescribing schemes so that third sector organisations can provide useful metrics and have access to a transparent assessment for referrals.

Future work should continue to explore and seek to understand different stakeholders’ perceptions of social prescribing and the issues of relevance to them, as social prescribing continues to be embedded nationally. Equally there is work to be done to support and develop links between service coordinators, referrers and providers to ensure social prescribing is understood and conceptualised coherently, that referrals are appropriate and the multitude of benefits of nature-based interventions are realised by a wide range of service users.
